# 3-Acetyl-1-(2,6-dimethyl­phen­yl)thio­urea

**DOI:** 10.1107/S1600536812029248

**Published:** 2012-07-04

**Authors:** Sharatha Kumar, Sabine Foro, B. Thimme Gowda

**Affiliations:** a Department of Chemistry, Mangalore University, Mangalagangotri 574 199, Mangalore, India; b Institute of Materials Science, Darmstadt University of Technology, Petersenstrasse 23, D-64287 Darmstadt, Germany

## Abstract

In the title compound, C_11_H_14_N_2_OS, the two N—H bonds are *anti* to each other and one of them is *anti* to the C=S and the other is *syn*. Further, the amide C=S and the C=O groups are *anti* to each other. The dihedral angle between the benzene ring and the side chain is 83.74 (5)°. An intra­molecular N—H⋯O hydrogen bond occurs. In the crystal, mol­ecules are linked into inversion dimers by pairs of N—H⋯S inter­actions.

## Related literature

For studies on the effects of substituents on the structures and other aspects of *N*-(ar­yl)-amides, see: Gowda *et al.* (2006 ▶); Shahwar *et al.* (2012 ▶), of *N*-(ar­yl)-methane­sulfonamides, see: Gowda *et al.* (2007 ▶) and of *N*-chloro­aryl­sulfonamides, see: Gowda *et al.* (2005 ▶); Shetty & Gowda (2004 ▶).
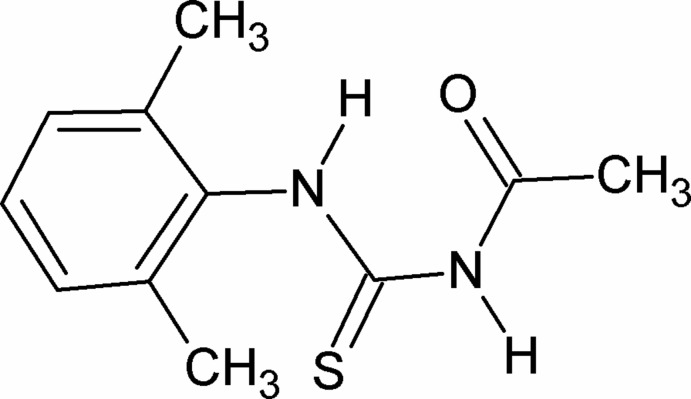


## Experimental

### 

#### Crystal data

C_11_H_14_N_2_OS
*M* *_r_* = 222.31
Triclinic, 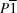

*a* = 8.008 (1) Å
*b* = 8.211 (1) Å
*c* = 10.037 (1) Å
α = 89.39 (1)°
β = 77.65 (1)°
γ = 64.71 (1)°
*V* = 580.47 (13) Å^3^
*Z* = 2
Mo *K*α radiation
μ = 0.26 mm^−1^
*T* = 295 K
0.30 × 0.20 × 0.08 mm

#### Data collection

Oxford Diffraction Xcalibur Sapphire CCD diffractometer
Absorption correction: multi-scan (*CrysAlis RED*; Oxford Diffraction, 2009 ▶) *T*_min_ = 0.928, *T*_max_ = 0.980
3904 measured reflections
2362 independent reflections
1858 reflections with *I* > 2σ(*I*)
*R*_int_ = 0.012

#### Refinement

*R*[*F* ^2^ > 2σ(*F* ^2^)] = 0.041
*wR*(*F* ^2^) = 0.115
*S* = 1.06
2362 reflections
145 parameters
2 restraints
H atoms treated by a mixture of independent and constrained refinement
Δρ_max_ = 0.21 e Å^−3^
Δρ_min_ = −0.31 e Å^−3^

### 

Data collection: *CrysAlis CCD* (Oxford Diffraction, 2009 ▶); cell refinement: *CrysAlis CCD*; data reduction: *CrysAlis RED* (Oxford Diffraction, 2009 ▶); program(s) used to solve structure: *SHELXS97* (Sheldrick, 2008 ▶); program(s) used to refine structure: *SHELXL97* (Sheldrick, 2008 ▶); molecular graphics: *PLATON* (Spek, 2009 ▶); software used to prepare material for publication: *SHELXL97*.

## Supplementary Material

Crystal structure: contains datablock(s) I, global. DOI: 10.1107/S1600536812029248/rk2369sup1.cif

Structure factors: contains datablock(s) I. DOI: 10.1107/S1600536812029248/rk2369Isup2.hkl

Supplementary material file. DOI: 10.1107/S1600536812029248/rk2369Isup3.cml

Additional supplementary materials:  crystallographic information; 3D view; checkCIF report

## Figures and Tables

**Table 1:** Hydrogen-bond geometry (Å, °)

*D*—H⋯*A*	*D*—H	H⋯*A*	*D*⋯*A*	*D*—H⋯*A*
N1—H1*N*⋯O1	0.84 (2)	1.97 (2)	2.658 (2)	138 (2)
N2—H2*N*⋯S1^i^	0.85 (2)	2.58 (2)	3.4012 (16)	164 (2)

Symmetry code: (i) 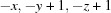.

**Fig. 1.**
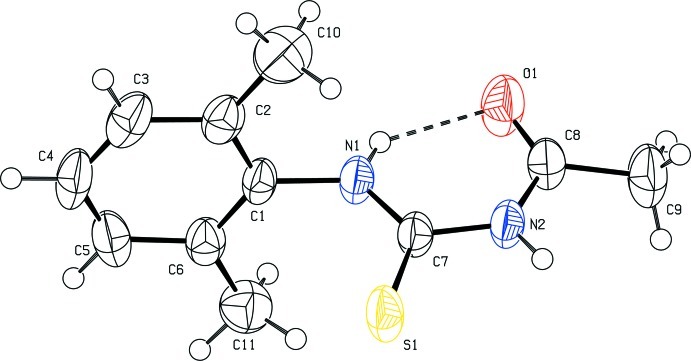
Molecular structure of the title compound, showing the atom labelling scheme. Displacement ellipsoids are drawn at the 50% probability level. H atom are presented as a small spheres of arbitrary radius.

**Fig. 2.**
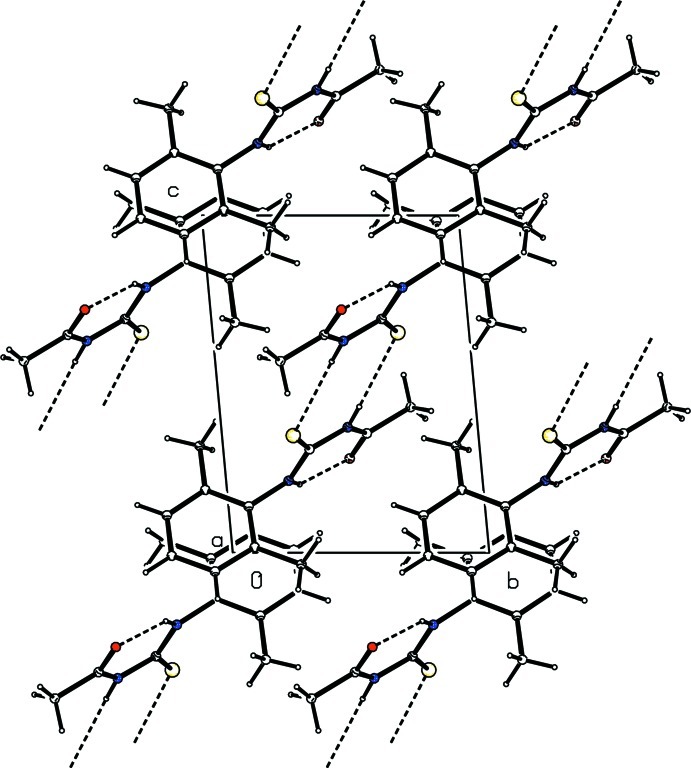
Molecular packing of the title compound with hydrogen bonding shown as dashed lines.

## supplementary crystallographic information

### Comment

Thiourea and its derivatives are widely used as precursors or intermediates in synthetic organic chemistry. They exhibit a wide variety of biological activities. As part of our studies on the substituent effects on the structures and other aspects of *N*-(aryl)-amides (Gowda *et al.*, 2006; Shahwar *et al.*, 2012); *N*-(aryl)-methanesulfonamides (Gowda *et al.*, 2007) and *N*-chloroarylsulfonamides (Gowda *et al.*, 2005; Shetty & Gowda, 2004), in the present work, the crystal structure of 3-acetyl-1-(2,6-dimethylphenyl)thiourea has been determined (Fig. 1).

The conformation of the two N–H bonds are *anti* to each other, and one of them is *anti* to the C═S and the other is *syn* in the urea moiety. Furthermore, the conformations of the amide C═S and the C═O are *anti* to each other, similar to the *anti* conformation observed in 3-acetyl-1-(2-methylphenyl)thiourea (Shahwar *et al.*, 2012).

The side chain is oriented itself with respect to the phenyl ring with the torsion angles of C2-C1-N1-C7 = 94.77 (22)° and C6-C1-N1-C7 = -87.11 (23)°. The dihedral angle between the phenyl ring and the side chain is 83.74 (5)°.

The structure shows intramolecular hydrogen bonding between the hydrogen atom of the NH attached to the phenyl ring and the amide oxygen. In the crystal, the molecules form inversion type dimers through N–H···S intermolecular classical hydrogen bonds (Table 1, Fig. 2).

### Experimental

The 3-acetyl-1-(2,6-dimethylphenyl)-thiourea was synthesized by adding a solution of acetyl chloride (0.10 mol) in acetone (30 ml) dropwise to a suspension of ammonium thiocyanate (0.10 mol) in acetone (30 ml). The reaction mixture was refluxed for 30 min. After cooling to room temperature, a solution of 2,6-dimethylaniline (0.10 mol) in acetone (10 ml) was added and refluxed for 3 h. The reaction mixture was poured into acidified cold water. The precipitated title compound was recrystallized to constant melting point from acetonitrile. The purity of the compound was checked and characterized by its IR spectrum. The characteristic absorptions observed are 3156.1 cm^-1^, 1698.1 cm^-1^, 1365.7 cm^-1^ and 710.4 cm^-1^ for the stretching bands of -N–H, -C═O, -C–N- and -C═S, respectively.

Prism like light yellow single crystals used in X-ray diffraction studies were grown in acetonitrile solution by slow evaporation of the solvent at room temperature.

### Refinement

The amino H atoms were freely refined with the N–H distances restrained to 0.86 (2)Å. H atoms bonded to C were positioned with idealized geometry using a riding model with the aromatic C–H = 0.93Å, methyl C–H = 0.96Å. All H atoms were refined with isotropic displacement parameters set at 1.2*U*_eq_(C-aromatic, N) and 1.5*U*_eq_(*C*-methyl) of the parent atom.

### Crystal data

**Table:**

C_11_H_14_N_2_OS	*Z* = 2
*M**_r_* = 222.31	*F*(000) = 236
Triclinic, *P*1	*D*_x_ = 1.272 Mg m^−^^3^
Hall symbol: -P 1	Mo *K*α radiation, λ = 0.71073 Å
*a* = 8.008 (1) Å	Cell parameters from 1747 reflections
*b* = 8.211 (1) Å	θ = 2.8–27.6°
*c* = 10.037 (1) Å	µ = 0.26 mm^−^^1^
α = 89.39 (1)°	*T* = 295 K
β = 77.65 (1)°	Prism, light yellow
γ = 64.71 (1)°	0.30 × 0.20 × 0.08 mm
*V* = 580.47 (13) Å^3^	

### Data collection

**Table:**

Oxford Diffraction Xcalibur Sapphire CCD diffractometer	2362 independent reflections
Radiation source: fine-focus sealed tube	1858 reflections with *I* > 2σ(*I*)
Graphite monochromator	*R*_int_ = 0.012
Rotation method data acquisition using ω and φ scans	θ_max_ = 26.4°, θ_min_ = 2.8°
Absorption correction: multi-scan (*CrysAlis RED*; Oxford Diffraction, 2009)	*h* = −9→6
*T*_min_ = 0.928, *T*_max_ = 0.980	*k* = −10→10
3904 measured reflections	*l* = −12→9

### Refinement

**Table:**

Refinement on *F*^2^	Primary atom site location: structure-invariant direct methods
Least-squares matrix: full	Secondary atom site location: difference Fourier map
*R*[*F*^2^ > 2σ(*F*^2^)] = 0.041	Hydrogen site location: inferred from neighbouring sites
*wR*(*F*^2^) = 0.115	H atoms treated by a mixture of independent and constrained refinement
*S* = 1.06	*w* = 1/[σ^2^(*F*_o_^2^) + (0.0515*P*)^2^ + 0.2097*P*] where *P* = (*F*_o_^2^ + 2*F*_c_^2^)/3
2362 reflections	(Δ/σ)_max_ = 0.016
145 parameters	Δρ_max_ = 0.21 e Å^−^^3^
2 restraints	Δρ_min_ = −0.31 e Å^−^^3^

### Special details

**Table:**

Geometry. All s.u.'s (except the s.u. in the dihedral angle between two l.s. planes) are estimated using the full covariance matrix. The cell s.u.'s are taken into account individually in the estimation of s.u.'s in distances, angles and torsion angles; correlations between s.u.'s in cell parameters are only used when they are defined by crystal symmetry. An approximate (isotropic) treatment of cell s.u.'s is used for estimating s.u.'s involving l.s. planes.
Refinement. Refinement of *F*^2^ against ALL reflections. The weighted *R*-factor *wR* and goodness of fit *S* are based on *F*^2^, conventional *R*-factors *R* are based on *F*, with *F* set to zero for negative *F*^2^. The threshold expression of *F*^2^ > σ(*F*^2^) is used only for calculating *R*-factors(gt) *etc*. and is not relevant to the choice of reflections for refinement. *R*-factors based on *F*^2^ are statistically about twice as large as those based on *F*, and *R*-factors based on ALL data will be even larger.

### Fractional atomic coordinates and isotropic or equivalent isotropic displacement parameters (Å^2^)

**Table:**

	*x*	*y*	*z*	*U*_iso_*/*U*_eq_	
C1	0.0904 (2)	0.0786 (2)	0.13742 (17)	0.0388 (4)	
C2	0.1028 (3)	−0.0846 (3)	0.1866 (2)	0.0467 (5)	
C3	0.2402 (3)	−0.2433 (3)	0.1110 (2)	0.0583 (6)	
H3	0.2520	−0.3542	0.1415	0.070*	
C4	0.3592 (3)	−0.2388 (3)	−0.0087 (2)	0.0605 (6)	
H4	0.4507	−0.3463	−0.0584	0.073*	
C5	0.3434 (3)	−0.0761 (3)	−0.0552 (2)	0.0553 (6)	
H5	0.4246	−0.0751	−0.1364	0.066*	
C6	0.2081 (3)	0.0875 (3)	0.01660 (19)	0.0441 (4)	
C7	−0.0262 (2)	0.3365 (2)	0.30688 (17)	0.0401 (4)	
C8	−0.3639 (3)	0.5655 (3)	0.35392 (19)	0.0476 (5)	
C9	−0.4949 (3)	0.7457 (3)	0.4319 (3)	0.0671 (7)	
H9A	−0.4325	0.8237	0.4212	0.080*	
H9B	−0.5285	0.7297	0.5272	0.080*	
H9C	−0.6077	0.7989	0.3972	0.080*	
C10	−0.0265 (4)	−0.0907 (4)	0.3167 (3)	0.0736 (7)	
H10A	−0.1540	−0.0437	0.3048	0.088*	
H10B	−0.0218	−0.0189	0.3894	0.088*	
H10C	0.0136	−0.2135	0.3393	0.088*	
C11	0.1920 (3)	0.2635 (3)	−0.0344 (2)	0.0621 (6)	
H11A	0.1901	0.3396	0.0385	0.075*	
H11B	0.0768	0.3217	−0.0656	0.075*	
H11C	0.2987	0.2425	−0.1087	0.075*	
N1	−0.0546 (2)	0.2439 (2)	0.21360 (16)	0.0434 (4)	
H1N	−0.166 (2)	0.286 (3)	0.203 (2)	0.052*	
N2	−0.1819 (2)	0.4932 (2)	0.37213 (16)	0.0446 (4)	
H2N	−0.158 (3)	0.546 (3)	0.4329 (19)	0.054*	
O1	−0.4147 (2)	0.4896 (2)	0.27946 (18)	0.0697 (5)	
S1	0.18238 (7)	0.27758 (8)	0.34744 (6)	0.0605 (2)	

### Atomic displacement parameters (Å^2^)

**Table:**

	*U*^11^	*U*^22^	*U*^33^	*U*^12^	*U*^13^	*U*^23^
C1	0.0349 (9)	0.0356 (9)	0.0395 (9)	−0.0070 (7)	−0.0135 (7)	−0.0102 (7)
C2	0.0511 (11)	0.0436 (11)	0.0458 (10)	−0.0171 (9)	−0.0189 (8)	−0.0028 (8)
C3	0.0619 (13)	0.0365 (11)	0.0731 (14)	−0.0107 (10)	−0.0315 (12)	−0.0045 (10)
C4	0.0471 (11)	0.0438 (12)	0.0716 (15)	0.0030 (9)	−0.0233 (11)	−0.0238 (10)
C5	0.0383 (10)	0.0656 (14)	0.0449 (11)	−0.0080 (9)	−0.0061 (8)	−0.0163 (10)
C6	0.0399 (9)	0.0452 (10)	0.0420 (10)	−0.0120 (8)	−0.0129 (8)	−0.0060 (8)
C7	0.0387 (9)	0.0384 (9)	0.0366 (9)	−0.0122 (8)	−0.0048 (7)	−0.0075 (7)
C8	0.0425 (10)	0.0437 (11)	0.0444 (10)	−0.0086 (8)	−0.0068 (8)	−0.0076 (8)
C9	0.0521 (12)	0.0519 (13)	0.0708 (15)	0.0008 (10)	−0.0109 (11)	−0.0173 (11)
C10	0.0924 (19)	0.0756 (17)	0.0596 (14)	−0.0442 (15)	−0.0147 (13)	0.0097 (12)
C11	0.0633 (14)	0.0621 (14)	0.0605 (13)	−0.0273 (11)	−0.0133 (11)	0.0067 (11)
N1	0.0352 (8)	0.0397 (9)	0.0463 (9)	−0.0069 (7)	−0.0106 (7)	−0.0145 (7)
N2	0.0404 (8)	0.0405 (8)	0.0437 (9)	−0.0100 (7)	−0.0068 (7)	−0.0148 (7)
O1	0.0475 (8)	0.0647 (10)	0.0810 (11)	−0.0048 (7)	−0.0238 (8)	−0.0261 (8)
S1	0.0389 (3)	0.0689 (4)	0.0590 (3)	−0.0087 (2)	−0.0118 (2)	−0.0315 (3)

### Geometric parameters (Å, º)

**Table:**

C1—C6	1.390 (3)	C8—O1	1.212 (2)
C1—C2	1.394 (3)	C8—N2	1.372 (3)
C1—N1	1.439 (2)	C8—C9	1.500 (3)
C2—C3	1.387 (3)	C9—H9A	0.9600
C2—C10	1.498 (3)	C9—H9B	0.9600
C3—C4	1.376 (3)	C9—H9C	0.9600
C3—H3	0.9300	C10—H10A	0.9600
C4—C5	1.373 (3)	C10—H10B	0.9600
C4—H4	0.9300	C10—H10C	0.9600
C5—C6	1.393 (3)	C11—H11A	0.9600
C5—H5	0.9300	C11—H11B	0.9600
C6—C11	1.490 (3)	C11—H11C	0.9600
C7—N1	1.327 (2)	N1—H1N	0.839 (15)
C7—N2	1.392 (2)	N2—H2N	0.848 (15)
C7—S1	1.6694 (19)		
			
C6—C1—C2	122.75 (16)	C8—C9—H9A	109.5
C6—C1—N1	119.14 (17)	C8—C9—H9B	109.5
C2—C1—N1	118.08 (16)	H9A—C9—H9B	109.5
C3—C2—C1	117.73 (19)	C8—C9—H9C	109.5
C3—C2—C10	120.5 (2)	H9A—C9—H9C	109.5
C1—C2—C10	121.76 (18)	H9B—C9—H9C	109.5
C4—C3—C2	120.8 (2)	C2—C10—H10A	109.5
C4—C3—H3	119.6	C2—C10—H10B	109.5
C2—C3—H3	119.6	H10A—C10—H10B	109.5
C5—C4—C3	120.25 (18)	C2—C10—H10C	109.5
C5—C4—H4	119.9	H10A—C10—H10C	109.5
C3—C4—H4	119.9	H10B—C10—H10C	109.5
C4—C5—C6	121.4 (2)	C6—C11—H11A	109.5
C4—C5—H5	119.3	C6—C11—H11B	109.5
C6—C5—H5	119.3	H11A—C11—H11B	109.5
C1—C6—C5	117.06 (19)	C6—C11—H11C	109.5
C1—C6—C11	121.87 (17)	H11A—C11—H11C	109.5
C5—C6—C11	121.07 (19)	H11B—C11—H11C	109.5
N1—C7—N2	116.66 (16)	C7—N1—C1	123.82 (15)
N1—C7—S1	124.10 (13)	C7—N1—H1N	115.6 (15)
N2—C7—S1	119.24 (13)	C1—N1—H1N	120.5 (15)
O1—C8—N2	122.32 (17)	C8—N2—C7	128.57 (16)
O1—C8—C9	122.46 (19)	C8—N2—H2N	118.1 (15)
N2—C8—C9	115.22 (18)	C7—N2—H2N	113.3 (15)
			
C6—C1—C2—C3	0.5 (3)	N1—C1—C6—C11	1.8 (3)
N1—C1—C2—C3	178.56 (16)	C4—C5—C6—C1	0.2 (3)
C6—C1—C2—C10	−179.58 (19)	C4—C5—C6—C11	179.9 (2)
N1—C1—C2—C10	−1.5 (3)	N2—C7—N1—C1	−179.56 (17)
C1—C2—C3—C4	−0.2 (3)	S1—C7—N1—C1	1.3 (3)
C10—C2—C3—C4	179.9 (2)	C6—C1—N1—C7	−87.1 (2)
C2—C3—C4—C5	−0.1 (3)	C2—C1—N1—C7	94.8 (2)
C3—C4—C5—C6	0.1 (3)	O1—C8—N2—C7	4.7 (3)
C2—C1—C6—C5	−0.5 (3)	C9—C8—N2—C7	−174.9 (2)
N1—C1—C6—C5	−178.51 (15)	N1—C7—N2—C8	0.6 (3)
C2—C1—C6—C11	179.82 (18)	S1—C7—N2—C8	179.79 (17)

### Hydrogen-bond geometry (Å, º)

**Table:**

*D*—H···*A*	*D*—H	H···*A*	*D*···*A*	*D*—H···*A*
N1—H1*N*···O1	0.84 (2)	1.97 (2)	2.658 (2)	138 (2)
N2—H2*N*···S1^i^	0.85 (2)	2.58 (2)	3.4012 (16)	164 (2)

Symmetry code: (i) −*x*, −*y*+1, −*z*+1.
